# Ca^2+^ tunnelling through the ER lumen as a mechanism for delivering Ca^2+^ entering via store‐operated Ca^2+^ channels to specific target sites

**DOI:** 10.1113/JP272772

**Published:** 2017-03-16

**Authors:** Ole H Petersen, Raphael Courjaret, Khaled Machaca

**Affiliations:** ^1^ MRC Group, School of Biosciences and Systems Immunity Research InstituteCardiff UniversityCardiffCF10 3AXUK; ^2^ Department of Physiology and BiophysicsWeill Cornell Medicine QatarEducation City, Qatar Foundation, PO Box 24144DohaQatar

**Keywords:** calcium activated chloride current, calcium entry, calcium signalling

## Abstract

Ca^2+^ signalling is perhaps the most universal and versatile mechanism regulating a wide range of cellular processes. Because of the many different calcium‐binding proteins distributed throughout cells, signalling precision requires localized rises in the cytosolic Ca^2+^ concentration. In electrically non‐excitable cells, for example epithelial cells, this is achieved by primary release of Ca^2+^ from the endoplasmic reticulum via Ca^2+^ release channels placed close to the physiological target. Because any rise in the cytosolic Ca^2+^ concentration activates Ca^2+^ extrusion, and in order for cells not to run out of Ca^2+^, there is a need for compensatory Ca^2+^ uptake from the extracellular fluid. This Ca^2+^ uptake occurs through a process known as store‐operated Ca^2+^ entry. Ideally Ca^2+^ entering the cell should not diffuse to the target site through the cytosol, as this would potentially activate undesirable processes. Ca^2+^ tunnelling through the lumen of the endoplasmic reticulum is a mechanism for delivering Ca^2+^ entering via store‐operated Ca^2+^ channels to specific target sites, and this process has been described in considerable detail in pancreatic acinar cells and oocytes. Here we review the most important evidence and present a generalized concept.

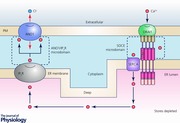

**Abbreviations**
ACh: acetylcholine
ANO1: anoctamin‐1
CaCC: Ca^2+^‐activated Cl^−^ channel
CCK: cholecystokinin
CRAC channel: Ca^2+^ release‐activated Ca^2+^ channel
ER: endoplasmic reticulum
IP_3_: inositol trisphosphate
IP_3_R: IP_3_ receptor
MCU: mitochondrial Ca^2+^ uniporter
NAADP: nicotinic acid adenine dinucleotide phosphate
NFAT: nuclear factor of activated T‐cells
PIP_2_: phosphatidyl inositol bisphosphate
PM: plasma membrane
PMCA: plasma membrane Ca^2+^‐activated ATPase
RyR: ryanodine receptor
SERCA: sarco/endoplasmic reticulum Ca^2+^ activated ATPase
SOAR: STIM1 Orai1‐activating region
SOCE: store‐operated Ca^2+^ entry
STIM: stromal interaction molecule
TMEM16A: transmembrane member 16A
TRP: transient receptor potential

## Introduction

**Figure 1:**
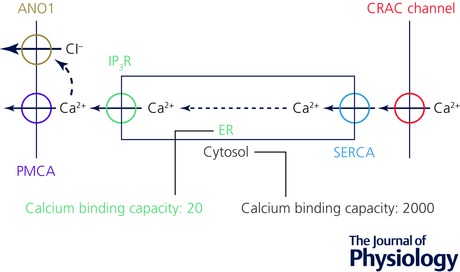
Schematic drawing of Ca^2+^ movement through pancreatic acinar cells Ca^2+^ moves from the extracellular solution (extreme right) via store‐operated CRAC channels, SERCA pumps, ER, IP_3_Rs and PMCA pumps into the acinar lumen (extreme left). Diffusion of Ca^2+^ from the base of the cell to the apical pole occurs quantitatively inside the ER rather than in the cytosol because of the much higher Ca^2+^ binding capacity in the cytosol than in the ER lumen. Ca^2+^ released from the ER in the apical part of the cell via IP_3_Rs activates Cl^−^ channels (ANO1) in the luminal acinar plasma membrane. ANO1, anoctamin‐1; CRAC channel, Ca^2+^ release‐activated Ca^2+^ channel; IP_3_R, inositol trisphosphate receptor; PMCA, plasma membrane Ca^2+^‐activated ATPase; SERCA, sarco/endoplasmic reticulum Ca^2+^ activated ATPase.

Effective and precise intracellular Ca^2+^ signalling depends on specific Ca^2+^ sensors and transport proteins expressed differentially on organelle and plasma membranes, as well as Ca^2+^ buffers with different affinities and kinetics in different cellular compartments (Petersen *et al*. 1994; Berridge, 2016). Because Ca^2+^ can interact with many potential cellular targets, signalling precision requires localized rises of the cytosolic [Ca^2+^] ([Ca^2+^]_i_) (Petersen *et al*. 1994; Petersen & Verkhratsky, 2016). In the nervous system, the extremely precise control of presynaptic neurotransmitter secretion depends on close co‐localization of voltage‐gated Ca^2+^ channels and the exocytotic machinery (Südhof, 2013). However, the target for the action of Ca^2+^ cannot always be very close to the site of Ca^2+^ entry. A prime example of such a scenario comes from the physiology of the pancreatic acinar cells, where Ca^2+^ entry occurs at the base of the cell, whereas the control of secretion has to take place at the opposite end of the cell at the apical pole (Petersen *et al*. 1994; Petersen & Tepikin, 2008). In these and many other electrically non‐excitable cells, which do not have voltage‐gated Ca^2+^ channels and do not fire action potentials (Petersen, 1992), the primary Ca^2+^ movement is from the endoplasmic reticulum (ER) into the cytosol (Nielsen & Petersen, 1972; Berridge, 2016) and this, in turn, triggers store‐operated Ca^2+^ entry (Putney, 1986; Petersen & Tepikin, 2008; Parekh, 2010). Diffusion of Ca^2+^ through the cytosol, from an entry site to a distant target, would potentially activate many inappropriate processes and a mechanism that could avoid this path would therefore be advantageous. Transport through an organelle, moving Ca^2+^ from its entry point to its target, would solve this problem (Fig. 1). The process of Ca^2+^ tunnelling through the ER was discovered in studies on pancreatic acinar cells carried out 20 years ago (Mogami *et al*. 1997). A similar process was later described in dopamine neurons (Choi *et al*. 2006) and the whole concept has more recently been generalized, based on experiments in oocytes (Courjaret & Machaca, 2014; Courjaret *et al*. 2016a). Furthermore, in a recent study (Kar *et al*. 2016), it has been shown that Ca^2+^ refilling of the nuclear envelope, after inositol trisphosphate (IP_3_)‐evoked Ca^2+^ release into the nucleoplasm through the inner nuclear membrane (Gerasimenko *et al*. 1995), depends on Ca^2+^ (entering via store‐operated Ca^2+^ channels) being tunnelled through the ER lumen directly into the nuclear envelope. In this article, we describe and review the most important evidence for Ca^2+^ tunnelling, primarily based on studies of pancreatic acinar cells and oocytes.

## Spatial and temporal features of Ca^2+^ signals

Specificity in Ca^2+^ signals is encoded in their spatial, temporal and amplitude features. These Ca^2+^ dynamics combine to activate a defined subset of Ca^2+^‐dependent downstream effectors to transduce the cellular response (Berridge *et al*. 2000, 2003). Spatially, Ca^2+^ signals are tightly regulated and are typically initiated by elementary events due to the opening of Ca^2+^ channels (intracellular or at the cell membrane). These elementary events due to the opening of one or a few channels can either remain localized resulting in Ca^2+^ signals in the microdomain around the channel(s), or coalesce through complex mechanisms into more global Ca^2+^ events that often encompass the entire cell (Berridge, 1997).

The extent and speed of Ca^2+^ movement is heavily influenced by the concentration and characteristics of the available Ca^2+^ buffers. It was shown many years ago, that adding a low affinity mobile Ca^2+^ buffer to the cytosol can profoundly change the timing and spatial extension of agonist‐elicited cytosolic Ca^2+^ signals (Petersen *et al*. 1991). The cytosolic Ca^2+^ buffering characteristics vary markedly between different cell types with, for example, a high level of relatively low mobility buffer in pancreatic acinar cells (Mogami *et al*. 1999), and a less restricted environment for Ca^2+^ diffusion in oocytes (Allbritton *et al*. 1992) and some nerve cells (Lin *et al*. 2017). Inevitably, [Ca^2+^] measurements using Ca^2+^‐sensitive fluorescent probes will be influenced by the Ca^2+^‐binding properties of the probes, so unless a careful analysis of the Ca^2+^ buffering situation has been carried out, as recently described by Lin *et al*. (2017), some caution with regard to interpreting quantitative results is called for.

In general, the diffusion of Ca^2+^ in the cytosol is always severely limited, as compared to movement in water, due to the relatively high buffering capacity. Some estimates indicate that free Ca^2+^ diffuses < 0.1 μm, and is free for ∼0.5 μs before it is buffered (Allbritton *et al*. 1992; Kasai & Petersen, 1994). At the mouth of an open Ca^2+^ channel and given the great concentration gradients across both the ER and plasma membrane, Ca^2+^ flow rapidly overwhelms the local buffering capacity resulting in a microdomain of high Ca^2+^ concentration in the order of 20–200 μm (Rizzuto & Pozzan, 2006). The spatial spread of these high Ca^2+^ microdomains is thus very tightly controlled, and is predicted based on theoretical modelling to be maintained within 20 nm of the channel (Simon & Llinas, 1985; Neher, 1998). Beyond the immediate point source of Ca^2+^ entry at the mouth of the channel, Ca^2+^ diffusion creates a downward gradient away from the channels that is thought to dissipate to the submicromolar range within 200 nm of the channel (Neher, 1998; Shuai & Parker, 2005; Demuro & Parker, 2006). This provides for an elegant mechanism to activate Ca^2+^‐dependent effectors that localize within the spatial spread of Ca^2+^ signals generated due to elementary Ca^2+^ events (Rizzuto & Pozzan, 2006; Parekh, 2008).

Global Ca^2+^ signals, in contrast, have a significantly broader spread on the order of 10–100 μm. This highlights a spatial gap between elementary and global Ca^2+^ signals, as there could be a physiological need to activate effectors that are not in the immediate vicinity of a Ca^2+^ channel without inducing a global Ca^2+^ rise that would activate a multitude of other signalling pathways. Although not discussed in details here, cells could maintain specificity in their Ca^2+^ signals despite the extent of their spatial spread by controlling their amplitude and frequency for example. Nonetheless, there are several examples that argue that Ca^2+^ signals activate effectors in the mid‐range, between the microdomain and global spatial extremes, with exquisite specificity. Nuclear factor of activated T‐cells (NFAT) activation in T‐cells in response to antigen stimulation (Dolmetsch *et al*. 1997; Rao *et al*. 1997), Ca^2+^‐activated K^+^ channels (Liu *et al*. 1998), and Ca^2+^‐activated Cl^−^ channels (Courjaret & Machaca, 2014) are examples of effectors activated specifically without localizing to the Ca^2+^ channel microdomain. It is, however, not clear mechanistically how cells would mediate such Ca^2+^ signals in the mid‐range without inducing a global Ca^2+^ rise. One possible mechanism is to induce a higher level or a more sustained Ca^2+^ flux through open channels to saturate the local buffering capacity, thus allowing the Ca^2+^ signal to spread further. This would be a perilous path, though, given the Ca^2+^ dependence of intracellular Ca^2+^ release channels, IP_3_ and ryanodine receptors, which could lead to Ca^2+^‐induced Ca^2+^ release and a global Ca^2+^ rise (Osipchuk *et al*. 1990; Bootman *et al*. 2002).

Ca^2+^ signalling in electrically non‐excitable cells is typically initiated downstream of agonist stimulation through the activation of a phospholipase C that hydrolyses phosphatidyl inositol bisphosphate (PIP_2_) at the plasma membrane and results in the production of IP_3_ and diacylglycerol. IP_3_ diffuses and gates open IP_3_ receptors at the ER membrane, releasing store Ca^2+^ to mediate the first phase of the Ca^2+^ signal. Should the Ca^2+^ release phase result in significant store depletion, it leads to the activation of Ca^2+^ influx at the plasma membrane through store‐operated Ca^2+^ entry (SOCE). SOCE is mediated by members of the stromal interaction molecule (STIM) and Orai family (Prakriya & Lewis, 2015). STIM1 is a single‐pass ER membrane protein with lumenal EF hands allowing it to sense the ER Ca^2+^ concentration (Liou *et al*. 2005; Roos *et al*. 2005). Store depletion results in STIM1 losing its lumenal bound Ca^2+^ leading to a conformational change in the protein and its clustering and translocation to ER–plasma membrane (PM) junctions that are 20 nm apart (Luik *et al*. 2006; Prakriya *et al*. 2006; Stathopulos *et al*. 2006; Vig *et al*. 2006; Wu *et al*. 2006; Yeromin *et al*. 2006; Liou *et al*. 2007). The close proximity of the ER and PM at these junctions allows STIM1 to span the distance and physically interact with Orai1 at the PM. Orai1 is a four transmembrane domain protein that forms a hexameric channel that is exquisitely Ca^2+^ selective (Hou *et al*. 2012). STIM1 clusters stabilize at ER–PM junctions initially through interaction of the poly‐lysine domain at the C‐terminal end of STIM1 with PIP_2_ in the PM. Activated STIM1 in response to store depletion exposes the STIM1 Orai1‐activating region (SOAR)/CRAC‐activating domain, which interacts with Orai1, traps it within the STIM1‐defined ER–PM junctions, and gates it open, thus allowing Ca^2+^ flow into the cell. SOCE activation not only results in store refilling but also shapes Ca^2+^ signal dynamics. There is therefore a tight functional link between IP_3_‐dependent Ca^2+^ release in response to agonist stimulation and Ca^2+^ influx through SOCE.

## Ca^2+^ tunnelling in pancreatic acinar cells

### The function of the acinar cells

The principal function of the exocrine pancreas is to deliver digestive enzymes to the intestine in order to break down food products, so that they can be absorbed into the circulation. The most important secretory cell in the exocrine pancreas is the acinar cell, which manufactures the inactive pro‐enzymes and stores them in zymogen granules. When enzyme delivery is required, the acinar cells receive a signal in the form of either the neurotransmitter acetylcholine (ACh; released from parasympathetic nerve endings) and/or the hormone cholecystokinin (CCK). Interaction with specific surface membrane receptors activates signal transduction mechanisms that generate intracellular Ca^2+^, liberating messengers – IP_3_ in the case of ACh and cyclic ADP‐ribose and nicotinic acid adenine dinucleotide phosphate (NAADP) in the case of CCK stimulation – thereby releasing Ca^2+^ from intracellular stores (Petersen & Tepikin, 2008). As a consequence of the depletion of intracellular Ca^2+^ stores, Ca^2+^‐permeable channels in the plasma membrane are opened allowing Ca^2+^ entry from the extracellular solution (Petersen & Tepikin, 2008).

Under physiological conditions the cytosolic Ca^2+^ concentration ([Ca^2+^]_i_) changes evoked by ACh or CCK consist of repetitive short‐lasting elevations mostly confined to the apical region (Kasai *et al*. 1993; Thorn *et al*. 1993), where the zymogen granules are concentrated. This local rise in [Ca^2+^]_i_ triggers exocytosis of the granule content (Maruyama & Petersen, 1994) as well as opening up Cl^−^ channels in the apical membrane and K^+^ channels in the apical part of the lateral membrane (Petersen & Maruyama, 1984; Petersen, 1992; Park *et al*. 2001b). These channel openings enable operation of the Na^+^/K^+^/2Cl^−^ co‐transporter as well as increased turnover of the Na^+^/K^+^ pump across the basolateral membrane (Petersen, 1992). The net result is uptake of Cl^−^ across the basolateral membrane and secretion of Cl^−^ across the apical membrane with Na^+^ following via a paracellular pathway through the leaky tight junctions. Water moves along with the salt both through the cell and through the so‐called tight junctions (Petersen, 1992). The Ca^2+^‐activated acinar fluid secretion is a vehicle for transport of the pro‐enzymes into the duct system, where an additional ductal fluid secretion of a secretin‐stimulated bicarbonate‐rich solution (Scratcherd *et al*. 1981; Lee *et al*. 2012) will help wash the pro‐enzymes into the gut where they become active digestive enzymes.

### The polarity of the acinar cells

The acinar cells secrete enzymes and fluid in one direction, namely into the lumen of the acinar unit, which is directly connected to the duct system and, therefore, these cells are highly polarized. The zymogen granules are in the apical part of the cells, whereas the nucleus – surrounded by densely packed rough ER – occupies the basolateral region. The apical membrane area is much smaller than the basolateral membrane area, but the final stage of secretion occurs exclusively through the apical membrane. The tight junctions, which are leaky in the case of the acinar epithelium, are placed close to the acinar lumen. Although the bulk of the ER is in the basolateral region, ER elements penetrate into the apical zymogen granule‐rich region all the way to the apical membrane (Gerasimenko *et al*. 2002).

With regard to the localization of the principal Ca^2+^‐activated ion channels in the plasma membrane of the acinar cells, we know that the Ca^2+^‐activated Cl^−^ channels, transmembrane member 16A (TMEM16A)/anoctamin‐1 (ANO1) (Lee *et al*. 2012), are exclusively present in the apical membrane (Park *et al*. 2001b), whereas the high‐conductance and voltage‐sensitive Ca^2+^‐activated K^+^ channels (present in the pig and human acinar cells) are found in the basolateral membrane (Maruyama *et al*. 1983; Petersen *et al*. 1985). Simultaneous patch clamp recording of Cl^−^ conductance and capacitance indicate that during normal physiological stimulus–secretion coupling, each local apical Ca^2+^ spike causes near‐synchronous (but see below) opening of Cl^−^ channels and exocytosis (Maruyama & Petersen, 1994).

With regard to Ca^2+^ transport across the plasma membrane, we know that Ca^2+^‐ATPase‐driven Ca^2+^ extrusion occurs mostly through the apical membrane (Belan *et al*. 1996), whereas store‐operated Ca^2+^ entry occurs through the basolateral membrane (Mogami *et al*. 1997; Park *et al*. 2001a; Lur *et al*. 2009).

### How Ca^2+^ entering through the basolateral plasma membrane allows Ca^2+^ signal generation near the apical membrane without passing through the cytosol

Although it has been known from the earliest days of work on stimulus–secretion coupling in pancreatic acinar cells that the initial Ca^2+^ signal generation evoked by stimulation with either ACh or CCK is due to release of Ca^2+^ from internal stores (Matthews *et al*. 1973; Petersen & Ueda, 1976), it has also been clear that supply of Ca^2+^ from the extracellular solution is essential for continuation of secretion (Petersen & Ueda, 1976). The reason for the extracellular Ca^2+^ requirement is that every rise in the cytosolic Ca^2+^ concentration ([Ca^2+^]_i_) inevitably activates Ca^2+^ pumps in the plasma membrane (plasma membrane Ca^2+^‐activated ATPase; PMCA) resulting in extrusion of Ca^2+^ (Tepikin *et al*. 1992), which then has to be compensated by Ca^2+^ entry, as otherwise the cell would gradually run out of Ca^2+^.

Agonist‐elicited cytosolic Ca^2+^ spiking, which is the normal physiological signal for secretion, requires a relatively high [Ca^2+^] in the lumen of the ER (Park *et al*. 2000). In experiments on isolated acinar cells where [Ca^2+^] changes in both the cytosol and the lumen of the ER were measured simultaneously, it could be shown that ACh evokes cytosolic Ca^2+^ spiking for several minutes in the absence of external Ca^2+^. However, spiking subsequently stops after only a relatively modest reduction of [Ca^2+^]_ER_ (Park *et al*. 2000). Thus, Ca^2+^ entry through store‐operated Ca^2+^ channels, refilling the ER store, is essential for the normal physiological function of the acinar cells.

The original concept of Ca^2+^ tunnelling through the ER lumen, from entry at the base of the cell to release near the apical membrane (Fig. 1), was based on experiments in which isolated acinar cells were kept in a Ca^2+^‐free solution with a patch pipette attached to the basal surface (Mogami *et al*. 1997). The patch pipette was filled with a Ca^2+^‐containing solution and Ca^2+^ entry across the membrane covered by the pipette tip could be regulated by controlling the pipette potential. After supra‐maximal ACh stimulation had emptied the intracellular stores during a period without Ca^2+^ entry (negative – retaining – potential in the pipette), ACh stimulation was discontinued and a period of Ca^2+^ entry was enabled by switching the pipette potential from negative to positive. No change was observed in [Ca^2+^]_i_ during this Ca^2+^ entry period, but after discontinuation of Ca^2+^ entry (switching the pipette voltage back to negative), a new period of ACh stimulation caused a local rise of [Ca^2+^]_i_ in the apical pole near the apical membrane, exactly as under normal conditions. A rise in [Ca^2+^]_i_ near the cell‐attached pipette during Ca^2+^ entry could only be observed when the ER Ca^2+^ pumps (sarco/endoplasmic reticulum Ca^2+^‐activated ATPase; SERCA) were arrested by thapsigargin (Thastrup *et al*. 1989). However, in this situation there was no sign of transfer of Ca^2+^ from the base to the apex, as ACh stimulation after a period of Ca^2+^ entry failed to elicit any Ca^2+^ release in the apical region (Mogami *et al*. 1997). As thapsigargin is a very selective blocker of SERCA pumps (Thastrup *et al*. 1989), the simplest explanation for the phenomenon observed, namely the non‐cytosolic transfer of Ca^2+^ across the cell from base to lumen, is movement through the ER lumen.

The ER Ca^2+^ tunnelling concept (Mogami *et al*. 1997) assumed that Ca^2+^ would move more easily within the lumen of the ER than in the cytosol (Fig. 1). The relatively low mobility of Ca^2+^ in the cytosol was demonstrated in the classical experiments of Baker & Crawford (1972) on axoplasm, in which it could be shown that radioactive Mg^2+^ moved much more quickly than radioactive Ca^2+^, and later confirmed by Allbritton *et al*. (1992). In the acinar cells, based on measurements of absolute calcium movements and changes in [Ca^2+^] in the cytosol and the ER, we estimated that the calcium binding capacity in the ER lumen is about 20 whereas in the cytosol it is about 1500–2000 (Mogami *et al*. 1999). Thus the mobility of Ca^2+^ in the ER lumen is very much higher than in the cytosol (Fig. 1).

The high mobility of Ca^2+^ in the ER lumen was demonstrated directly by experiments in which changes in [Ca^2+^]_ER_ at various locations in the ER could be monitored after a highly localized uncaging of caged Ca^2+^ in the ER lumen (Park *et al*. 2000). These experiments showed that after a local Ca^2+^ uncaging event, rises in [Ca^2+^]_ER_ were observed quickly over considerable distances (more than 10 μm away from the site of uncaging) and that the whole of the ER was re‐equilibrated with regard to [Ca^2+^]_ER_ within a few seconds (less than the time interval between cytosolic Ca^2+^ spikes during physiological Ca^2+^ signalling) (Park *et al*. 2000; Petersen *et al*. 2001).

### Movement of Ca^2+^ from the extracellular fluid into the ER lumen at the base

**Figure 2:**
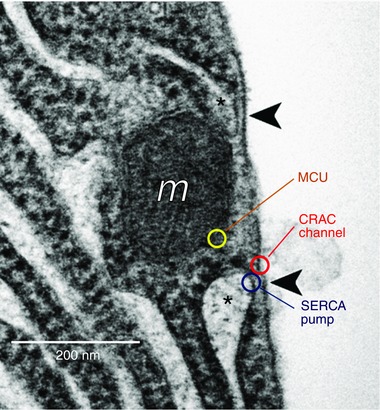
Location of key organelles and molecules involved in Ca^2+^ uptake at the base of pancreatic acinar cells EM picture of basal area of a pancreatic acinar cell showing areas in which the ER is devoid of ribosomes and comes very close to the plasma membrane (indicated with asterisk). This arrangement allows molecular interaction between STIM in the ER membrane and CRAC channels in the plasma membrane. A mitochondrion (*m*) is also seen near the plasma membrane. Arrowheads signpost ER–plasma membrane junctions where Ca^2+^ entry can take place. Indicative locations of the three key Ca^2+^ transporters at the base of the cell are shown. MCU, mitochondrial Ca^2+^ uniporter. Adapted from Lur *et al*. (2009).

Early work on perfused submandibular glands showed that ACh‐evoked intracellular Ca^2+^ release was followed, after a delay, by Ca^2+^ influx into the gland cells from the perfusion fluid (Nielsen & Petersen, 1972). Later, work on isolated pancreatic acinar cells showed more precisely that the Ca^2+^ entry, following the ACh‐evoked immediate (< 0.5 s) apical Ca^2+^ release, occurred through the basolateral membrane after a delay of about 6–7 s (Toescu & Petersen, 1995). A few years later it was shown that it is possible to refill the emptied ER with Ca^2+^ flowing into the cell from a point source at the base of the cell (Mogami *et al*. 1997). Like in many other cell types, store‐operated Ca^2+^ entry is mediated by translocation of STIM to puncta near the plasma membrane, which in the pancreatic acinar cells are specifically located at the basolateral part of the cell (Lur *et al*. 2009). However, in these cells there is a specific challenge for this process, as the ER is of the rough type due to the presence of ribosomes. The size of ribosomes is such that it would not allow the close molecular interaction between STIM in the ER membrane and Ca^2+^ channels in the plasma membrane that is necessary for channel activation. However, it turns out that there are small areas of the otherwise rough ER that are devoid of ribosomes, allowing these parts to come very close to the plasma membrane (Lur *et al*. 2009) (Fig. 2).

The biophysical nature of the Ca^2+^ entry process in the pancreatic acinar cells was not clarified until recently (Gerasimenko *et al*. 2013), when patch clamp whole‐cell current recording studies showed that the inward Ca^2+^ current evoked by ER store Ca^2+^ depletion has characteristics very similar to the Ca^2+^ release activated Ca^2+^ (CRAC) current previously discovered in immune cells (Hoth & Penner, 1992; Feske, 2007; Parekh, 2010), and could be blocked by a specific CRAC channel inhibitor (Gerasimenko *et al*. 2013). During Ca^2+^ refilling of the ER, for example after ACh‐evoked emptying of the store, there is no measurable increase in the cytosolic Ca^2+^ concentration near the Ca^2+^ entry channels although, as mentioned above, it is possible to observe a rise in the cytosolic [Ca^2+^] during Ca^2+^ entry if the SERCA pumps in the ER have been arrested by thapsigargin (Mogami *et al*. 1997). In that case there is also a clear increase in the [Ca^2+^] in the mitochondria (mitochondrial Ca^2+^ uptake being mediated by the mitochondrial Ca^2+^ uniporter; MCU; De Stefani *et al*., 2011, 2016) placed very close to the basolateral membrane (Park *et al*. 2001a) (Fig. 2). Ca^2+^ uptake into these peripheral mitochondria is functionally important as it will increase ATP production (De Stefani *et al*. 2016) locally, thereby fuelling the SERCA pumps. It would therefore appear that the crucial molecules, involved in the process of moving Ca^2+^ from the extracellular fluid into the ER, namely CRAC channels, SERCA pumps and the MCU, are localized very close together in the basolateral part of the cell during SOCE (Fig. 2).

### Movement of Ca^2+^ from the ER into the apical cytosol where activation of exocytosis and Cl^−^ channels occurs

**Figure 3:**
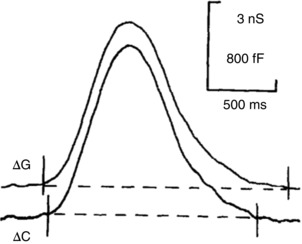
Ca^2+^‐activated secretory events at the apical pole of pancreatic acinar cells Simultaneous recording of changes in Cl^−^ conductance (Δ*G*) and capacitance (Δ*C*) during a single IP_3_‐elicited Ca^2+^ spike (part of a train of spikes evoked by continuous intracellular IP_3_ infusion) shows the similar timing and trend of both events with a slight delay in the capacitance increase as compared to the rise of the Cl^−^ conductance. It is also seen that the Cl^−^ conductance increase outlasts the period of increased capacitance. From Maruyama & Petersen (1994).

The rise in [Ca^2+^]_i_, evoked by either ACh or CCK stimulation, always starts in the apical part of the cell, close to the apical membrane (Kasai & Augustine, 1990; Kasai *et al*. 1993; Thorn *et al*. 1993; Cancela *et al*. 2000) and, at near‐physiological intensities of stimulation, the rise is mostly confined to the apical region (Kasai *et al*. 1993; Thorn *et al*. 1993), due to the perigranular mitochondrial firewall (Tinel *et al*. 1999; Park *et al*. 2001a). Even under conditions where muscarinic receptor activation occurs exclusively within a small region at the base of the cell (uncaging of caged carbachol in a cell‐attached patch pipette), the rise in [Ca^2+^]_i_ always starts near the apical membrane (Ashby *et al*. 2003). Close comparison of the time course of the increases in Cl^−^ conductance and capacitance (indicative of fusion between zymogen granules and apical plasma membrane) during individual apical Ca^2+^ spikes shows (Fig. 3) that Cl^−^ channels are activated slightly earlier than the start of exocytosis and that the Cl^−^ conductance increase slightly outlasts the period of increased capacitance (Maruyama & Petersen, 1994). This could be explained either by the Cl^−^ channels being located closer to the ER Ca^2+^ release channels than the sites of exocytosis, or by the Cl^−^ channels being more sensitive to the local [Ca^2+^]_i_ changes than the exocytosis machinery.

Ca^2+^ spiking, induced by stimulation with either ACh or CCK, is abolished by blockade of IP_3_ receptors (IP_3_Rs) (Wakui *et al*. 1990), but also by blockade of ryanodine receptors (RyRs) (Cancela *et al*. 2000). Since Ca^2+^ spiking can also be elicited by intracellular Ca^2+^ infusion (Osipchuk *et al*. 1990; Wakui *et al*. 1990), it is probably due to interactive Ca^2+^‐induced Ca^2+^ release, involving both IP_3_Rs and RyRs. The mechanisms by which ACh and CCK initiate apical Ca^2+^ signal generation are different. In the case of ACh stimulation, there is IP_3_ generation due to phospholipase C activation whereas in the case of physiological CCK stimulation (low picomolar CCK concentration), there is primary generation of NAADP (Yamasaki *et al*. 2005). Thus blockade of NAADP receptors inhibits CCK‐ but not ACh‐elicited Ca^2+^ spiking (Cancela *et al*. 2000; Gerasimenko *et al*. 2015). In spite of these mechanistic differences, the measurable Ca^2+^ signal progression from the initiation site near the apical membrane towards the perigranular mitochondrial belt is quantitatively very similar in both cases (Cancela *et al*. 2000). This indicates that the initial trigger Ca^2+^ release is so small and so local that it is not observable with current technology. The local apical Ca^2+^ release that actually activates the Cl^−^ channels in the apical membrane and the exocytotic enzyme release through the apical plasma membrane is therefore most likely the result of the final co‐activation of IP_3_Rs and RyRs triggered by the initial Ca^2+^ release from either IP_3_Rs or NAADP‐sensitive two‐pore channels (Gerasimenko *et al*. 2015).

## Ca^2+^ tunnelling supports mid‐range Ca^2+^ signalling in the *Xenopus* oocyte

### The *Xenopus* oocyte as an experimental model system to study Ca^2+^ signalling

The frog oocyte has long been a favoured model system to study Ca^2+^ signalling and has contributed significantly to our understanding of basic Ca^2+^ signalling mechanisms, including elementary Ca^2+^ release events, Ca^2+^ waves, fertilization‐specific Ca^2+^ signals, biophysical properties of the IP_3_ receptor, and remodelling of Ca^2+^ signalling during the cell cycle (Lechleiter & Clapham, 1992; Sun *et al*. 1998; Bugrim *et al*. 2003; Foskett *et al*. 2007; Machaca, 2007). Several features make the oocyte an attractive model system to study these various aspects of Ca^2+^ signalling. The oocyte is large (∼1.2 mm in diameter) allowing for easy spatial resolution of Ca^2+^ dynamics, which becomes particularly important for studies focused on the generation and propagation of Ca^2+^ waves, and elementary Ca^2+^ release events because their large spatial footprint in the oocyte makes them more amenable to investigation. The size of the oocyte also favours biochemical analyses and importantly linking them directly to Ca^2+^ signalling and other cell physiological processes at the single cell level (Machaca & Haun, 2002). Another unique advantage of the oocyte is the stage of the cell cycle oocytes transition through with two physiologically defined arrest points at prophase I and metaphase II of meiosis. Oocytes are arrested in prophase I at the G2/M transition of the cell cycle in a G2‐like state, which is the typical stage in which they have been used as an experimental model. However, physiologically oocytes transition to metaphase of meiosis II in preparation for fertilization. This well‐regulated progression through M‐phase provides a distinctive window into the cell division phase of the cell cycle that is transient and asynchronized in other systems such as mitosis in mammalian cells, making it more difficult to study. Another additional benefit of the oocyte is the relative simplicity of its Ca^2+^ signalling toolkit compared to other cells. The frog oocyte has a limited well defined complement of Ca^2+^ channels and transporters, significantly less complex than most mammalian cells (Machaca, 2007).

Further simplifying Ca^2+^ signalling studies in the frog oocyte is the endogenous expression of Ca^2+^‐activated Cl^−^ channels (CaCCs), which are critical for oocyte biology and fertilization as they contribute significantly to the maintenance and regulation of the membrane potential. The Ca^2+^ transient generated at fertilization is initially localized at the site of sperm entry but gradually sweeps across the entire egg in the form of a Ca^2+^ release wave, which activates CaCCs and depolarizes the cell to prevent polyspermy (Machaca *et al*. 2001). This so called ‘fast electrical block’ to polyspermy in *Xenopus* is due to the fact that sperm–egg fusion is voltage sensitive in this species (Jaffe *et al*. 1983; Goul‐Somero & Jaffe, 1984). The molecular entity underlying the CaCC in the frog oocyte has been identified as anoctamin 1 (Ano1) or TMEM16A (Schroeder *et al*. 2008; Yang *et al*. 2008). The biophysical properties of the *Xenopus* oocyte CaCCs have been well characterized both for the endogenous current (Kuruma & Hartzell, 1998, 2000; Machaca & Hartzell, 1998, 1999; Callamaras & Parker, 2000), overexpressed Ano1 in the oocyte (Courjaret *et al*. 2016b), and heterologously expressed *Xenopus* Ano1 in the axolotl oocyte (Schroeder *et al*. 2008). In the oocyte, CaCC senses sub‐cell membrane changes in Ca^2+^ concentration in real time and with high fidelity, whether this Ca^2+^ is released from the ER or flows from the extracellular space through channels in the plasma membrane (Machaca & Hartzell, 1999). As such multiple studies have used the endogenous CaCC to monitor complex Ca^2+^ dynamics mediated by endogenous or heterologously expressed Ca^2+‐^permeable channels, such as ionotropic receptors (Kuruma & Hartzell, 1998), voltage‐gated Ca^2+^ channels (Zhou *et al*. 2004), transient receptor potential (TRP) channels (Courjaret *et al*. 2013) and SOCE channels (Courjaret & Machaca, 2016).

### Mid‐range Ca^2+^ signalling

**Figure 4:**
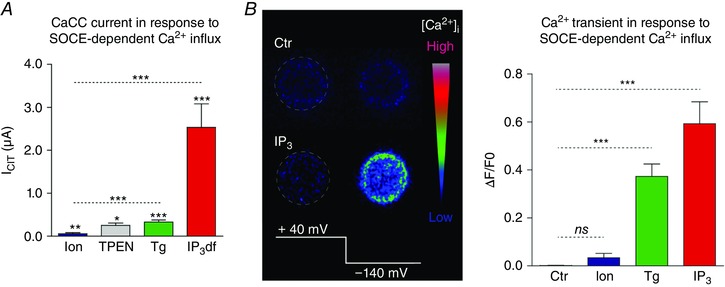
Differential response of Ca^2+^‐activated Cl^−^ channels to modes of store depletion in the oocyte *A*, amplitudes of current through CaCC following store depletion with different agents: ionomycin, TPEN, thapsigargin (Tg), non‐hydrolyzable IP_3_ (IP_3_df). *B*, left, intracellular Ca^2+^ transient monitored by confocal microscopy in a voltage‐clamped oocyte using Oregon green BAPTA‐1 under control conditions (Ctr) or following IP_3_ injection. Ca^2+^ influx through SOCE was stimulated by hyperpolarizing the cell to −140 mV. Right, summary of the intracellular Ca^2+^ rise induced by the hyperpolarizing pulse to −140 mV when intracellular stores were depleted with ionomycin (Ion), thapsigargin (Tg) or IP_3_. Adapted from Courjaret and Machaca (2014).

**Figure 5:**
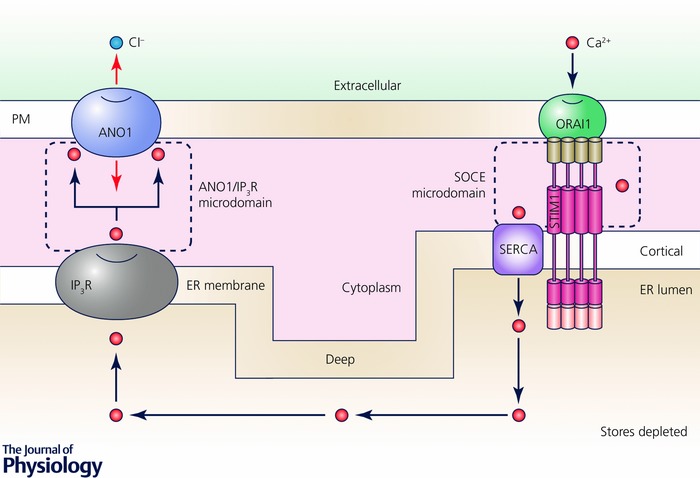
Model of Ca^2+^ tunnelling Cartoon depicting the mechanism of Ca^2+^ tunnelling downstream of Ca^2+^ store depletion and SOCE activation. Two distinct separate microdomains are at play. The SOCE microdomain, defined by STIM1‐Orai1 interactions, mediates Ca^2+^ flow from the extracellular space into the narrow ER–PM junction where it is taken up into the ER through the action of SERCA. The second domain is defined by IP_3_R release sites that localize close to CaCCs to selectively activate them. Ca^2+^ flows from the extracellular space through Orai1 resulting in a localized Ca^2+^ microdomain that is spatially limited due to rapid uptake of Ca^2+^ by SERCA into the ER lumen. With open IP_3_Rs, Ca^2+^ flowing into the ER leaks out through IP_3_R to activate CaCC.

While studying the activation properties of CaCCs in the frog oocyte in response to various Ca^2+^ mobilizing agents, we noticed that CaCCs are stimulated to significantly higher levels when stores are depleted with IP_3_ as compared to other mobilizing agents that deplete Ca^2+^ stores by distinct mechanisms of action, including ionomycin, (*N*,*N*,*N*,*N*‐tetrakis(2‐pyridylmethyl)‐ethylenediamine (TPEN) and thapsigargin (Fig. 4) (Courjaret & Machaca, 2014). While IP_3_ replicates the physiological situation, ionomycin, a Ca^2+^ ionophore with preferential insertion in the ER membrane (Morgan & Jacob, 1994), empties Ca^2+^ stores rapidly and induces SOCE in the absence of activation of IP_3_ receptors (IP_3_R). TPEN is a transition metal chelator with low Ca^2+^ affinity that is freely membrane permeant and chelates high lumenal ER Ca^2+^, thus simulating Ca^2+^ store depletion and inducing SOCE (Hofer *et al*. 1998), again in the absence of IP_3_R activation. Thapsigargin is an irreversible, specific blocker of the endoplasmic reticulum Ca^2+^‐ATPase (SERCA) (Thastrup *et al*. 1989) that leads to store depletion due to an inherent constitutive Ca^2+^ leak pathway from the ER. Therefore, blocking SERCA, the primary ER Ca^2+^ refilling pathway leads to store depletion, although with slower kinetics than other aforementioned Ca^2+^ mobilizing agents. Given the differing mechanisms by which these agents induce SOCE, it was not clear why IP_3_ leads to a significantly higher induction of current through CaCCs. We ruled out an increased Ca^2+^ influx through SOCE under these various conditions (Courjaret & Machaca, 2014). Furthermore, we showed that Ca^2+^ transients in the cortical region of the oocyte were of similar amplitude when SOCE was stimulated with IP_3_ or thapsigargin, but of much smaller amplitude with ionomycin (Fig. 4 *B*) (Courjaret & Machaca, 2014). Given that measuring SOCE current reveals similar flow of Ca^2+^ into the cell, these data show that when SERCA is active, Ca^2+^ flowing through SOCE channels is immediately taken up by SERCA into the ER lumen, thus preventing its diffusion into the cortical region. When SERCA is blocked with thapsigargin, this pathway is inhibited allowing SOCE to flood the cell cortex with Ca^2+^. However, this does not explain why cortical Ca^2+^ is high during the Ca^2+^ influx phase when stores are depleted with IP_3_ (Fig. 4 *B*). Various approaches to block the IP_3_R or SERCA were used to show that the high CaCC current in response to SOCE when IP_3_ is present requires active IP_3_Rs (Courjaret & Machaca, 2014). This led to the model outlined in Fig. 5, where Ca^2+^ flowing through SOCE channels is taken up within the SOCE microdomain by SERCA into the ER lumen and then released through open IP_3_Rs at a distal site to specifically activate CaCCs, thus leading to high current levels specifically in response to SOCE (Fig. 5). This model matches the Ca^2+^ tunnelling mechanism discussed above in pancreatic acinar cells although the timing and functional links between the various components of the pathway diverge to meet the cell's physiological needs.

**Figure 6:**
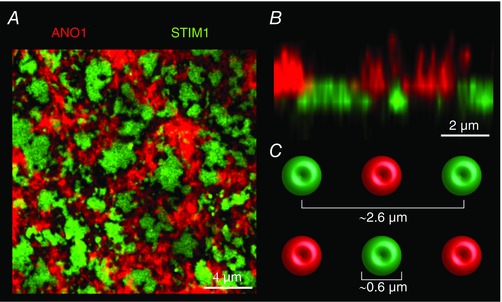
Spatial reorganization of the Ca^2+^ signalling machinery and effectors in response to Ca^2+^ store depletion *A*, confocal imaging of *Xenopus* Ano1 tagged with mCherry (red) and of the ER Ca^2+^ sensor STIM1 tagged with green fluorescent protein (green) after store depletion induced by IP_3_ injection. The example is an extreme situation where STIM1 forms large fused clusters that exclude the CaCC Ano1. *B*, orthogonal reconstruction of a section of the oocyte in *A*. The microvilli covered by Ano1 are clearly visible as well as the separation between the SOCE domains and the domains painted by Ano1. *C*, dimensions and spatial spread of the SOCE clusters as measured in Courjaret and Machaca (2014).

Careful co‐localization experiments in the oocyte confirm this model and show that Orai1, STIM1 and SERCA localize to the SOCE clusters at ER–PM junctions, thereby creating a specialized Ca^2+^ handling domain that favours Ca^2+^ influx into the cytoplasm through Orai1 and uptake into the ER through SERCA (Courjaret & Machaca, 2014). Importantly, in the case of CaCC as a downstream Ca^2+^ effector, store depletion is associated with a dramatic remodelling of the Ca^2+^ signalling machinery. STIM1, Orai1 and SERCA localize to SOCE clusters at ER–PM junctions. In contrast, Ano1 is excluded from these junctions and localizes to other areas of the plasma membrane (Fig. 6). At rest, Ano1 is evenly distributed throughout the cell membrane of the oocyte, including the dense brush of microvilli where it serves an additional scaffolding function and regulates microvilli length (Courjaret *et al*. 2016b). Store depletion, while concentrating STIM1, Orai1 and to a lesser extend SERCA into the SOCE clusters, excludes Ano1 resulting in the patchy separation illustrated in Fig. 6. In other words, there is little to no co‐localization of the Ca^2+^ entry source (SOCE) and of the Ca^2+^ effector (CaCC), and the distance to be covered by Ca^2+^ ions in the cytoplasm from the mouth of the Orai channel to the CaCC is incompatible with a diffusion mechanism given the speed of activation of CaCCs and the measured size and distribution of SOCE puncta as compared to CaCC‐rich membrane domains (Fig. 6). To overcome the diffusion barrier and reach the CaCCs, Ca^2+^ ions transit through the ER and are released at the target spot by IP_3_R. This spatial reorganization of the Ca^2+^ signalling machinery mediating SOCE and Ca^2+^ release results in the delivery of Ca^2+^ flowing into the cell through SOCE to a distal effector, CaCC, without inducing a global Ca^2+^ rise or having to contend with the limiting cytoplasmic Ca^2+^ diffusion. This signalling module allows for the transport of information carried by Ca^2+^ influx across distances that exceed the SOCE microdomain and are in the micrometre range or ‘mid‐range’ between elementary and global signals.

Therefore, with SERCA active, the cytoplasmic Ca^2+^ transient within SOCE puncta at ER–PM junctions is transient and very localized, with the majority of the Ca^2+^ flux going into the ER and then leaking out at distal sites through IP_3_Rs to activate effectors with high specificity and efficiency. This is consistent with the small flux through Orai1 channels. Localization of SERCA to the STIM/Orai cluster is not restricted to *Xenopus* oocytes and has been reported in other cell types (Jousset *et al*. 2007; Sampieri *et al*. 2009; Alonso *et al*. 2012; Hogan, 2015). Therefore, following store depletion and the activation of SOCE, a pump–leak balance develops at the ER membrane with a point source pump pathway mediated by Orai1–STIM1–SERCA that is physically localized at SOCE puncta, and a leak pathway through open IP_3_Rs at distal sites to activate effectors such as CaCC (Fig. 5). We have previously proposed the term ‘Ca^2+^ teleporting’ to borrow an analogy from science fiction (Fort, 1931), to suggest rapid transport of Ca^2+^ through the ER lumen given the fact that ER stores are never fully depleted of Ca^2+^ (Courjaret *et al*. 2016a). Although a single Ca^2+^ ion is obviously not instantaneously traversing that distance through the ER lumen, the term teleporting nicely reflects the transfer of Ca^2+^ from the SOCE entry sites to CaCCs in a directed fashion to modulate CaCC current. In fact a direct physical interaction has been reported between the IP_3_receptor and Ano1 in neurons (Jin *et al*. 2013; Jin *et al*. 2014); whether a similar interaction exists in the oocyte remains unknown.

In addition to modulating the spatial aspects of Ca^2+^ signals and effector activation, Ca^2+^ tunnelling also modulates the temporal aspects of Ca^2+^ signals by favouring tonic over oscillatory Ca^2+^ signalling (Courjaret *et al*. 2016a). When Ca^2+^ stores are relatively full, IP_3_ production favours Ca^2+^ oscillations resulting in repetitive transient Ca^2+^ signals. In contrast, when SOCE is fully activated with depleted Ca^2+^ store, Ca^2+^ tunnelling mediates pump–leak balance at the ER membrane that favours tonic sustained Ca^2+^ signalling but inhibiting Ca^2+^ oscillations (Courjaret *et al*. 2016a). In this case Ca^2+^ tunnelling through the ER lumen targets the IP_3_ receptor itself and modulates its properties to favour tonic rather than oscillatory signalling. Therefore, Ca^2+^ tunnelling not only modulates the spatial aspects of Ca^2+^ signalling, but also affects the temporal features of Ca^2+^ signals in the same cell. This has significant implications for encoding specific cellular responses downstream of SOCE using Ca^2+^ tunnelling.

## Is Ca^2+^ tunnelling active in other cells?

The Ca^2+^ tunnelling system is clearly functional in pancreatic acinar cells and in the frog oocyte. Both of these cell types are highly specialized for a specific function, secretion in the acinar cell and fertilization and support of early development in the oocyte. Therefore, the question arises as to whether this Ca^2+^ signalling mode is ubiquitous or unique to some highly specialized cell types. We currently do not know the answer to this question, but several arguments support the conclusion that this Ca^2+^ signalling modality is widespread. First, SOCE is ubiquitous in non‐excitable cells and present in excitable cells as well. SOCE is physiologically linked to IP_3_ receptor activation downstream of agonist stimulation. Therefore, the entire machinery supporting Ca^2+^ tunnelling is present. The spatial remodelling of the Ca^2+^ signalling machinery in response to store depletion outlined in the oocyte system translates and has been described in other cells as well. Furthermore, the IP_3_R has been shown to directly link to CaCC, a defined effector for Ca^2+^ tunnelling. Conceptually, the Ca^2+^ tunnelling mechanism is quite attractive as it allows for specific signalling to effectors through the SOCE pathway without inducing a global Ca^2+^ rise in the cytosol and without the need to localize multiple effectors into the physically limited space defined by ER–PM junctions where the SOCE machinery localizes.

In principle, Ca^2+^ tunnelling through the ER should be present in all cell types as it would seem unlikely that SERCA pumps and Ca^2+^ release channels should be exactly co‐localized. Therefore Ca^2+^ would always tunnel a bit between Ca^2+^ uptake and release sites. The length of the effective tunnel would vary between cell types depending on their function. The effectiveness and speed of Ca^2+^ tunnelling would depend critically on the concentrations of Ca^2+^ buffers in the ER lumen, their mobility as well as their binding and dissociation rate constants. In addition, it would depend on the degree of depletion of ER Ca^2+^ stores. In the pancreatic acinar cells, it has been shown directly that there are only minor reductions in [Ca^2+^]_ER_ during physiological stimulation (Park *et al*. 2000). In general, it is unlikely that there would be a need for complete depletion of ER Ca^2+^ stores before SOCE is activated. This is indeed the case in the frog oocyte, where IP_3_‐dependent release of Ca^2+^ from the ER fully activates SOCE without emptying the stores completely (Courjaret *et al*. 2016a).

In the ER lumen of the pancreatic acinar cells, the movement of Ca^2+^ immediately after localized uncaging of caged Ca^2+^ (following maximal ACh‐induced Ca^2+^ release) has been directly monitored. The rate of rise of [Ca^2+^]_ER_ decreases, as expected for a diffusional process, with increasing distance from the uncaging site (Park *et al*. 2000). At a distance of 10 μm from the uncaging site, the peak [Ca^2+^]_ER_ occurs ∼2.5 s later than at the uncaging site itself. Complete re‐equilibration of [Ca^2+^] in the whole of the ER is attained 6–8 s after the uncaging event (Park *et al*. 2000). These data underestimate the speed of Ca^2+^ movement in the ER lumen under physiological conditions, because of the necessity of first having to evoke maximal release of Ca^2+^ from the ER in order to obtain a clear local increase in [Ca^2+^]_ER_ upon Ca^2+^ uncaging. The free buffer concentration in these experiments (Park *et al*. 2000) would therefore have been higher than under more physiological conditions, where many of the buffers would already have been saturated with Ca^2+^.

One can readily postulate a long list of potential effectors that could be targeted by Ca^2+^ tunnelling with the most obvious being Ca^2+^‐regulated ion channels located at the plasma membrane such as CaCCs, Ca^2+^‐activated K^+^ channels (Liu *et al*. 1998), other integral membrane proteins such as adenylate cyclases (Halls & Cooper, 2011), and Ca^2+^ sensitive enzymes anchored at the plasma membrane through *A‐kinase anchor proteins* such as protein kinase C and phosphatase 2B (Esseltine & Scott, 2013). Ca^2+^ tunnelling effectors are likely to localize in the immediate vicinity of the release site, the IP_3_R, and this can include virtually all the downstream effectors of the IP_3_R that have been recently reviewed (Prole & Taylor, 2016). In the cytosol, organelles can also be a target for Ca^2+^ tunnelling, including lysosomes, nuclei, vesicles and mitochondria that can all localize next to IP_3_Rs. Mitochondria are of particular interest given their intimate interaction with SOCE and the localization of IP_3_R to ER‐mitochondria junctions (Parekh, 2003).

Currently there are few validated targets of Ca^2+^ tunnelling including CaCCs, Ca^2+^‐activated K^+^ channels, secretion in acinar cells, and the IP_3_R itself where we have shown that Ca^2+^ tunnelling can modulate IP_3_R activity switching it from a mode that favours Ca^2+^ oscillations to one that favours tonic Ca^2+^ signals (Courjaret *et al*. 2016a). There are also hints in the literature of potential additional effectors of Ca^2+^ tunnelling. In a human salivary gland cell line, the direct activation of the Ca^2+^‐activated K^+^ channel by SOCE is limited by the fast buffering of Ca^2+^ below the plasma membrane and can be restored when the ER Ca^2+^ pump is inhibited by thapsigargin. When SOCE and IP_3_ receptors are simultaneously activated (by stimulating muscarinic receptors with carbachol), Ca^2+^‐sensitive K^+^ channels are strongly activated, supporting the idea that SOCE ‘fuels’ the IP_3_ receptors when the stores are empty to provide an efficient activation of the K^+^ channel (Liu *et al*. 1998).

## Conclusion

Herein we focus on findings from two distinctive specialized cell types, the pancreatic acinar cell and the frog oocyte, that led to proposing a novel model of Ca^2+^ signalling that we refer to as Ca^2+^ tunnelling. In pancreatic acinar cells, Ca^2+^ tunnelling allows the transport of Ca^2+^ flowing from the basolateral membrane to support transepithelial fluid transport and secretion of digestive enzymes. The tunnelling of Ca^2+^ through the ER lumen circumvents the slow diffusion of Ca^2+^ through the highly buffered cytosol and importantly delivers Ca^2+^ to effectors in the apical membrane without inducing a global [Ca^2+^]_i_ rise, which would undoubtedly activate multiple other Ca^2+^‐dependent processes. In oocytes, Ca^2+^ tunnelling specifically and efficiently activates CaCCs downstream of SOCE without inducing a global Ca^2+^ rise. This activation occurs spatially in the mid‐range broader than the Ca^2+^ microdomain but more contained than a global [Ca^2+^]_i_ rise. This again eludes the need for Ca^2+^ to diffuse long distances in the highly buffered cytosol and avoids a global [Ca^2+^]_i_ rise while allowing the activation of a specific effector, CaCC, downstream of SOCE. In addition, Ca^2+^ tunnelling in the oocyte modulates the spatial features of Ca^2+^ signals favouring a tonic signal while inhibiting Ca^2+^ oscillations by acting on the IP_3_R itself, in this case as a downstream effector.

Of note is the mechanism underlying Ca^2+^ tunnelling with SOCE forming the Ca^2+^ entry pathway that fuels the whole process. Ca^2+^ entering the cell within the SOCE microdomain is unlikely to diffuse beyond the microdomain due both to the cytoplasmic Ca^2+^ buffering and also to the rapid uptake into the ER lumen through the action of SERCA. This is somewhat reminiscent of the capacitative Ca^2+^ entry model originally proposed by Jim Putney (Putney, 1986), where it was postulated that Ca^2+^ enters the cell directly from the extracellular space into the ER lumen. Although it is now clear that this is not the case, the limited diffusion of Ca^2+^ beyond the SOCE microdomain and the rapid uptake of Ca^2+^ flowing through SOCE into the ER lumen argue that a significant proportion of the signalling downstream of SOCE occurs through Ca^2+^ tunnelling.

Interestingly, the molecular mechanisms underlying Ca^2+^ tunnelling in acinar cells and oocytes are analogous. The machinery mediating Ca^2+^ tunnelling encompasses STIM1 and Orai1 (SOCE), the SERCA pump and the IP_3_R. Store depletion stabilizes the STIM1–Orai1 puncta at ER–PM junctions thus providing the source for Ca^2+^ entry from the extracellular space. Ca^2+^ flowing through SOCE channels is taken up by the SERCA pump into the ER lumen preventing its diffusion out of the SOCE microdomain. In turn ER Ca^2+^ is released through IP_3_Rs thus delivering it to the appropriate effectors (secretion, CaCC, IP_3_R) with high efficiency and specificity. The Ca^2+^ tunnelling machinery has been adapted to very different cell physiological needs in the oocyte as compared to the pancreatic acinar cell. In the oocyte it modulates resting membrane potential and the temporal features of Ca^2+^ signals, whereas in the acinar cell it drives enzyme secretion and fluid flow. Given that the molecular machinery underlying Ca^2+^ tunnelling is ubiquitous, it is likely that this pathway is involved in Ca^2+^ signalling in a plethora of other physiological functions. The remarkable functional link between SOCE, SERCA and IP_3_R conscripted to allow Ca^2+^ tunnelling results in the delivery of Ca^2+^ to effectors that could easily be missed experimentally and interpreted as signalling downstream of SOCE directly. It is therefore likely that Ca^2+^ tunnelling activates additional cell physiological events that remain to be defined.

## Additional information

### Competing interests

None declared.

### Author contributions

All authors have approved the final version of the manuscript and agree to be accountable for all aspects of the work. All persons designated as authors qualify for authorship, and all those who qualify for authorship are listed.

### Funding

O.H.P. is a Medical Research Council Professor (G19/22/2).
